# Impact of a decision-making aid for suspected urinary tract infections on antibiotic overuse in nursing homes

**DOI:** 10.1186/s12877-016-0255-9

**Published:** 2016-04-15

**Authors:** Darcy K. McMaughan, Obioma Nwaiwu, Hongwei Zhao, Elizabeth Frentzel, David Mehr, Sara Imanpour, Steven Garfinkel, Charles D. Phillips

**Affiliations:** Texas A&M University, College Station, USA; American Institutes for Research, Washington, D.C, USA; University of Missouri School of Medicine, Columbia, USA; Department of Health Policy & Management, School of Public Health, 1266 TAMU, College Station, TX 77843 USA

**Keywords:** Nursing home, Antibiotic stewardship, Urinary tract infection, Asymptomatic bacteriuria, Antibiotics

## Abstract

**Background:**

Antibiotics are highly utilized in nursing homes. The aim of the study was to test the effectiveness of a decision-making aid for urinary tract infection management on reducing antibiotic prescriptions for suspected bacteriuria in the urine without symptoms, known as asymptomatic bacteriuria (ASB) in twelve nursing homes in Texas.

**Method:**

A pre- and post-test with comparison group design was used. The data was collected through retrospective chart review. The study sample included 669 antibiotic prescriptions for suspected urinary tract infections ordered for 547 nursing home residents. The main measurement for the outcome variable was whether an antibiotic was prescribed for suspected urinary tract infections with no symptoms present.

**Results:**

Most of the prescriptions for antibiotics UTIs were written without documented symptoms – thus for asymptomatic bacteuria (ASB) (71 % during the pre-intervention period). Exposure to the decision-making aid decreased the number of prescriptions written for ASB (from 78 % to 65 % in the low-intensity homes and from 65 % to 57 % in the high-intensity homes), and decreased odds of a prescription being written for ASB (OR = 0.63, 95 % CI = 0.25 – 1.60 for low-intensity homes; OR = 0.79, 95 % CI = 0.33 – 1.88 for high-intensity homes). The odds of a prescription being written for ASB decreased significantly in homes that succeeded in implementing the decision-making aid (OR = 0.35, 95 % CI = 0.16–0.76), compared to homes with no fidelity.

**Conclusions:**

The decision-making aid improved antibiotic stewardship in nursing homes.

**Electronic supplementary material:**

The online version of this article (doi:10.1186/s12877-016-0255-9) contains supplementary material, which is available to authorized users.

## Background

Antibiotics are highly utilized in nursing homes. Up to 70 percent of nursing home residents may receive at least one antimicrobial agent a year [1, 2]. Similarly, [3] found that 47 % to 79 % of the nursing home residents receive at least on course of antibiotics per year [3]. High rates of antibiotic use make nursing homes a breeding ground for antibiotic-resistant bacteria, from Methicillin-resistant Staphylococcus aureus (MRSA) to resistant Clostridium difficile infections (CDI) [4, 5]. The emergence of multidrug resistant organisms in nursing homes and consequent spread to the community combined with other negative outcomes of antibiotic overuse, such as adverse drug events, hospital admissions, and higher health care costs, calls for optimizing antibiotic stewardship in nursing homes [4].

One avenue for reducing the overall rate of antibiotic prescriptions is to reduce the number of “inappropriate” antibiotic prescriptions. However, determining “appropriateness” of antibiotic use in nursing homes is challenging. In the nursing home setting antibiotic courses are often started empirically due to the limited availability of diagnostics. Even so, the general consensus is that inappropriate antibiotic use occurs frequently in nursing homes. Examples of potentially inappropriate practices in nursing homes include prescribing antibiotics as a prophylaxis, prescribing without a clear description of the source of the infection, and in the case of urinary tract infections (UTIs), prescribing antibiotics in the absence of specific symptoms indicating the presence of a UTI [6, 7]. Urinary tract infections are the most common nursing home infection, making proper treatment essential to nursing home quality of care [8, 9]. Up to one-third of prescriptions for suspected urinary tract infections in nursing home residents are for asymptomatic patients with bacteriuria (bacteria in the urine) [2]. This practice of prescribing antibiotics for bacteriuria in the absence of outward symptoms is possibly influenced by abnormal urinalyses rather than physical symptoms [10]. Bacteriuria without symptoms, known as asymptomatic bacteriuria (ASB), is common in nursing home residents, with prevalence as high as 50 percent – and is often related to changes in the functionality of the bladder and urinary tract associated with aging and comorbid conditions [11, 12]. There are consistent findings indicating that treating residents for bacteriuria without symptoms is not beneficial, and possibly harmful [13, 14]. The potential for large numbers of unnecessary antibiotic prescriptions in nursing homes makes this setting ideal for implementing an intervention aimed at reducing antibiotic use for ASB and reducing the overall rate of antibiotic prescriptions.

In this study we examine the effect of a decision-making aid on antibiotic stewardship in nursing homes. This aid was created through several workgroup meetings with technical experts from the Texas Department of Disability and Aging, nursing home administrators, and infection control specialists; and finalized with a usability test and a small scale trial in several Texas nursing homes. Results of the small scale trial can be found elsewhere [15]. The resulting decision-making aid built directly upon current nursing home processes, practice, and regulatory requirements. It centered upon recommendations from two clinical practice guidelines and follows the SBAR (Situation, Background, Analysis, and Recommendation) format [http://www.ahrq.gov/professionals/education/curriculum-tools/teamstepps/index.html]. SBAR is designed to promote a structured conversation between nurses and physicians to ensure that all the information required for decision making is conveyed in an expected order. SBAR is a standard component of the Agency for Healthcare Research and Quality’s TeamSTEPPS® patient safety model [2] [http://www.ahrq.gov/professionals/education/curriculum-tools/teamstepps/index.html] [16]. The aid focused on determining the presence or absence of physical symptoms of a UTI based on the High and the Loeb criteria, which are best practices model for antibiotic prescribing in nursing homes; and clinical practice guidelines from the Infectious Disease Society of America, and offered alternatives to antibiotic use when those symptoms were not present (Additional file 1) [16].

We hypothesized that:

- The decision-making aid would be effective at reducing antibiotic prescriptions for residents with asymptomatic bacteuria.
- The effectiveness of the decision-making aid would be contingent on amount of training nursing homes received on using the aid, and resident characteristics.

## Methods

### Study design

The design was a pre- and post-test with comparison with a six month pre-intervention period and a six month post-intervention period. Twelve nursing homes were assigned purposively to three groups (decision-making aid with high intensity training, decision-making aid with low intensity training, and comparison), with four homes per group. IRB approval has been obtained through Texas A&M University Institutional Review Board for human subjects (IRB number: 2009–0862). Before inclusion all participants provided written consent to participate in the study. High intensity homes received training in using the decision making aid twice during the intervention period, and received active technical support. Low intensity homes were trained on the decision making aid once, and received passive technical support only in response to requests. Low and high intensity home refers to the level of training and assistance received by nursing home residence. Active technical support consisted of trainers asking nursing homes if they experienced any issues with the decision making aid. Passive technical support consisted of trainers offering assistance only if requested by the nursing home. All training and technical support were provided by research nurses familiar with nursing home operations. The research nurses were further asked, at the end of the intervention period, to assess the level of each home’s fidelity to the intervention and intended use of the decision making aid. Project team members also conducted process evaluations at the end of the intervention period with nursing home staff and administrators. Data from the research nurses, in combination with data from the process evaluation interviews, were used to rank the project homes by level of fidelity to the intervention. High fidelity homes used the decision making aid for each instance of a suspected UTI. Low fidelity homes rarely (less than 25 % of suspected UTI cases) used the decision making aid. A post-hoc analysis, which grouped the nursing homes by fidelity to the decision-making aid, was conducted to determine whether variability in fidelity revealed the “true” effect of the decision-making aid. This resulted in two groups for the post-hoc analysis– homes that actually implemented the decision-making aid (fidelity group with four homes) and homes that did not implement the decision-making aid (no fidelity group with eight homes including the four control homes).

### Data

Data were collected monthly in all 12 Texas nursing homes for 6 months prior to introducing the decision-making aid and for 6 months following the introduction of the decision-making aid. The data were collected by clinically trained nurse consultants who routinely work with nursing homes. From March 2011 through February 2012, residents receiving an antibiotic for a suspected UTI were identified through retrospective chart reviews of the study homes’ infection logs, which facilities are required to maintain under federal regulations [56 FR 48876, Sept. 26, 1991, as amended at 57 FR 43925, Sept. 23, 1992]. Each of the listed UTIs were associated with an antibiotic prescription (with very few exceptions). As a result, the infection logs essentially represented antibiotic treatment logs. The research team collected data on resident characteristics at admission and information from the most recent quarterly Minimum Data Set (MDS) assessments [17]. The final dataset contained prescriptions written for suspected UTIs during the study time period.

### Measurement

#### Dependent variable

At the facility level, we considered the aggregate rates of overall antibiotic prescriptions for ASB. At the prescription level, the dependent variable was a binary variable indicating whether an antibiotic was prescribed in the absence of physical symptoms associated with a UTI (yes/no) –essentially, was the prescription ordered for asymptomatic bacteuria. These symptoms were based on two previously published consensus criteria: the Loeb criteria and the High criteria [2, 16]. Medical records were reviewed to abstract any of the following signs and symptoms from the criteria:

- acute dysuria,
- fever (>37.9 °C [100 °F]) or 1.5 °C [2.4 °F] increase above baseline temperature),
- new or worsening urgency, frequency, or incontinence,
- suprapubic pain,
- gross hematuria,
- costovertebral angle (flank) tenderness,
- rigors, and
- delirium (recent and abrupt change in mental status)

#### Independent variables

Resident characteristics associated with each antibiotics prescription were obtained using resident’s admission MDS and the most recent MDS completed prior to the date of the first suspected UTI. From this data, variables were created to determine if resident characteristics are associated with prescriptions for ASB. The variables included age, race/ethnicity, gender, and urinary incontinence. In addition, information on physical functioning and communication status were used to construct variables for level of performance of activities of daily living (an ADL hierarchy scale) [18] and the ability to verbally communicate symptoms related to UTIs. Age was categorized into three groups (75 and younger, 76 to 85, and 86 and older). Since the vast majority of residents were non-Hispanic whites, the race/ethnicity variable was dichotomized as non-Hispanic White and Other. The model also included the variable capturing time period (pre and post-test). The most important independent variable is the treatment group (high-intensity, low intensity, comparison). In the post-hoc analysis the model also included a variable representing nursing home level of fidelity to the decision-making aid.

### Statistical analyses

Data were analyzed using SAS 9.2. The analyses were intended to identify the effect of the decision-making aid on the odds of a prescription being written for ASB after controlling for resident characteristics.

Descriptive analyses were provided for the 12 nursing homes and 547 residents who received antibiotic orders for suspected UTIs during the study time period. Chi-square tests and one way ANOVA analysis were performed to test for statistically significant differences on resident characteristics among treatment groups. Aggregate rates of overall antibiotic prescriptions for ASB by different treatment groups were also calculated. Finally, a multivariate analysis was performed on the 669 prescriptions written for 547 residents. The unit of analysis for this multivariate modeling was the prescription. Due to the correlated nature of the prescription data, in that more than one prescription could be ‘nested’ within a resident, and prescriptions from the same resident home are also more or less “similar”, a mixed effect logistic regression model was used to estimate the effects of covariates on the likelihood of a prescription being written for ASB. Thus the dependence between observations due to ‘nesting’ of prescriptions was statistically adjusted using the GLIMMIX procedure in SAS, with nursing homes and resident identifiers as nested random effects. The independent variables mentioned earlier, together with the interaction of treatment and study period (pre vs post), were included in the model as fixed effects. The association between each independent variable and a prescription being written for ASB was expressed as odds ratio estimates with 95 % confidence intervals.

During a post-implementation process evaluation, it became apparent that some nursing homes implemented the tool, and some did not. There was a very stark difference in level of implementation, with homes either using the tool for each infection noted in the infection log, or never using the tool/using the tool for less than 25 % of infections. For the purpose of the post-hoc analysis, those home which implemented but never used the tool or used the tool with less than 25 % of infections were designated as ‘low fidelity’. The remaining homes used the tool for each infection. This post-hoc analysis included the entire post-intervention period.

In the post-hoc analysis, we compared aggregates rates of prescriptions for suspected UTI for homes groups according to fidelity (implementers vs. non-implementers) and used the GLIMMIX procedure to fit a regression model with fidelity as the main experimental variable. Fidelity to the decision making aid was determined through process evaluations conducted at the conclusion of the study. Of the four homes designated as having fidelity to the decision-making aid, three were from the high intensity training group and one was from the low intensity group. Of the eight homes designated as having no fidelity to the aid, one home was from the high-intensity group, three were from the low-intensity group, and four were from the comparison group. A mixed effect logistic regression model was fit to the data using similar fixed effects and random effects as in the main analysis, except substituting treatment group (high-intensity, low intensity, comparison) with the fidelity variable (implementers vs. non-implementers).

### Sample size consideration

The primary goal of the analysis is to compare the rate of correct use of antibiotics in the intervention groups with the comparison group. Power is the probability of rejecting the null hypothesis when the null hypothesis is wrong. In order to find the accurate measure of testing to reject the null hypothesis the power calculation was conducted. Based on the results (Table 1) a sample size of 110 in each group is required when the p-value is 0.05 and we have 80 % power to reject the null hypothesis.

**Table 1:** Nursing home characteristics of 12 Texas nursing homes

Characteristic	Comparison homes (*N* = 4)	Lower intensity intervention (*N* = 4)	Higher intensity intervention (*N* = 4)	*p* value
For profit *%(n*)	75 %	100 %	75 %	0.0000
Number of attending physicians *range*	3 – 8	1 – 15	2 – 12	
mean(SD)	5.8 (2.6)	7.0 (5.9)	5.3 (4.8)	0.0601
Average rate of antibiotic prescription per 1,000 resident bed days per month *range*	4.33 – 10.69	3.95 – 13.43	3.78 – 12.92	
mean (SD)	1.8 (0.59)	2.2 (2.3)	2.3 (2.4)	0.2461
Total bed days per month *range*	1550 – 3096	1144 – 3019	1603 – 3026	
mean (SD)	2133 (439)	2325 (609)	2358 (424)	0.0104
Total bed capacity *range*	78 – 120	88 – 120	103 – 120	
mean (SD)	95 (17.8)	97(15.4)	109.3(7.8)	<0.0001

## Results

### Home and resident characteristics

Tables 1 and 2 present data on the 12 participating nursing homes and residents who received antibiotics for a suspected UTI in the pre-intervention period. Home characteristics were consistent with the profile of a “typical” sample of US nursing homes, with a bed size between 78 and 120 beds, and a greater number of for-profit ownerships. Within each group, the rate of antibiotic prescriptions for suspected urinary tract infections ranged from approximately 4 to between 11 and 13 per 1,000 bed days of care. The number of active physicians also varied considerably. Some homes had only one to three physicians responsible for residents, while others had more than a dozen. In general, the residents included in the study fit the profile of the average nursing home resident in the United States [18]. Most of the residents were white, female, of older age, and experiencing some level of urinary incontinence/cognitive impairment/limitations in activities of daily living. Chi-square tests and one-way ANOVA tests showed that the average rate of antibiotic prescription per 1,000 resident bed days per month and the average number of physicians attending to patients in each home were not statistical significantly different between homes; however the total bed days per month, the home ownership status, and the total bed capacities differed between homes.

**Table 2:** Characteristics of residents with prescriptions for suspected UTI (*N* = 547)

Characteristic	Comparison homes (*N* = 4)	Lower intensity intervention (*N* = 4)	Higher intensity intervention (*N* = 4)
Age distribution			
≤75 *%*	20.39	29.41	22.40
76–85 *%*	34.87	29.90	33.33
86+ *%*	44.74	40.69	44.27
Female *%*	74.83	76.47	75.00
White *%*	88.08	85.29	86.98
Urinary incontinence *%*			
Always incontinent	21.85	24.02	26.04
Frequently incontinent	27.15	18.63	23.96
Occasionally incontinent	16.56	11.27	20.83
Never incontinent	17.22	29.41	18.75
Not rated	17.22	16.67	10.42
Catheterized *%*	16.67	16.67	10.82
ADL hierarchy (0–6) *mean (SD)*	2.19 (1.10)	2.38 (1.31)	2.22 (1.19)
Ability to communicate (0–3) *mean (SD)*	0.79 (0.98)	0.64 (0.89)	1.18 (0.84)

#### Aggregate rates of antibiotic prescriptions

Table 3 provides the percentages of prescriptions written for suspected ASB among all prescriptions for suspected UTIs during the entire study. The majority of the prescriptions were not associated with documented symptoms (71 %) during the pre-intervention period. The proportion of prescriptions for ASB increased slightly in the comparison homes and declined in both the low- (78 % to 65 %) and high-intensity decision-making aid sites (65 % to 57 %) from the pre to the post-intervention time periods. The eight homes that did not implement the decision-making aid with any meaningful degree of fidelity (“no-fidelity” group) showed almost no change (70 % to 69 %) from the pre- to the post-intervention period in the proportion of prescriptions written for ASB. The proportion of prescriptions for ASB in the four fidelity homes dropped from 73 percent to 49 percent, a reduction of one third.

**Table 3:** Percentage of prescriptions for asymptomatic bacteuria among all prescriptions in 12 Texas Nursing Home, broken down by treatment and fidelity groups

	Preintervention [*N* = 355]	Postintervention [*N* = 314]
Comparison homes	69.00	71.59
Low intensity homes	78.15	64.66
High intensity homes	65.44	57.27
Fidelity (post hoc)	73.15	49.35
No fidelity (post hoc)	69.64	68.78
Total	70.70	64.00

### Multivariate analysis

Table 4 presents results from a mixed effect logistic regression model using whether a prescription was written for ASB as the outcome, and the original treatment assignment as the main covariate. Both homes and residents were initially included as random effects. However, a likelihood ratio test indicated that a random effect for residents was not necessary, so only home was the random effect in the final model. The odds (from pre to post) of a prescription being written for ASB is lower in the low-intensity homes and in the high-intensity homes than in the comparison homes (OR = 0.63, 95 % CI = 0.25 – 1.60 for low-intensity group; OR = 0.79, 95 % CI = 0.33 – 1.88 for high-intensity group). However, neither effect reaches the traditional levels required for statistical significance. The only resident characteristics associated with antibiotic prescribing were incontinence and ability to communicate. Incontinence increased the odds of a prescription being written in the absence of symptoms (OR = 1.93, 95 % CI = 1.05–3.56 for “always incontinent”). A higher level of communication ability also increased the odds of a prescription being written in the absence of symptoms (OR = 1.26, 95 % CI = 1.00–1.59).

**Table 4:** Results from a mixed effect logistic regression model on the likelihood of ASB – using original treatment assignment as a covariate

	Odds ratio	95 % CI	p
Fixed effect			
High intensity training	0.77	0.32–1.86	0.57
Low intensity training	1.19	0.47–3.01	0.71
Age			
≤75 vs 86+	1.09	0.69–1.73	0.72
76–85 vs 86+	0.96	0.64–1.44	0.86
Female	0.84	0.54–1.30	0.43
White	1.11	0.64–1.95	0.71
Urinary incontinence ^a^			
Always incontinent vs continent	1.93	1.05–3.56	0.04
Frequently incontinent vs continent	1.36	0.78–2.38	0.28
Occasionally incontinent vs continent	1.16	0.65–2.09	0.61
Catheterized ^a^	1.04	0.48–2.24	0.92
ADL hierarchy (0–6)	1.07	0.89–1.27	0.47
Ability to communicate (0–3)	1.26	1.00–1.59	0.05
Post vs Pre	0.99	0.51–1.94	0.98
Interaction between time period and training			
Pre/post and high intensity^b^	0.79	0.33–1.88	0.59
Pre/post and low intensity^b^	0.63	0.25–1.60	0.33
Random Effect	Variance	St. Error	
Home	0.2105	0.1304	

^a^During the study time period the MDS 3.0 was implemented. During implementation nursing homes were given leeway in documenting status. This resulted in a high number of missing observations for variables such as urinary incontinence and presence of catheter. Sensitivity analyses were performed to determine the overall effect on the models; the models remained robust

^b^These interaction terms reflect the difference in changes from pre to post comparing treatment groups to the control group, so they are our main interest

In the post-hoc multivariate analysis (Table 5) of fidelity to treatment, results indicated that after controlling for differences in resident characteristics, the likelihood of a prescription being written for ASB decreased significantly in the homes that implemented the decision-making aid (OR = 0.35, 95 % CI = 0.16–0.76), compared to the homes with no fidelity. Again, both incontinence and communication ability increased the odds of a prescription being written in the absence of symptoms (OR = 1.96, 95 % CI = 1.05–3.61 for “always incontinent”; OR = 1.26, 95 % CI = 1.00–1.60).

**Table 5:** Results from a mixed effect logistic regression model on the likelihood of ASB – using fidelity to treatment as a covariate

	Odds ratio	95 % CI	*P*
Fixed Effect			
Fidelity	1.14	0.51–2.56	0.75
Age			
≤75 vs 86+	1.09	0.69–1.73	0.74
76–85 vs 86+	0.96	0.64–1.44	0.84
Female	0.83	0.54–1.29	0.40
White	1.13	0.64–2.00	0.67
Urinary incontinence ^a^			
Always incontinent vs continent	1.96	1.05–3.61	0.03
Frequently incontinent vs continent	1.34	0.77–2.35	0.30
Occasionally incontinent vs continent	1.13	0.63–2.03	0.69
Catheterized ^a^	1.07	0.50–2.29	0.86
ADL hierarchy (0–6)	1.08	0.90–1.28	0.40
Ability to communicate (0–3)	1.26	1.00–1.60	0.05
Post vs Pre	1.05	0.70–1.60	0.81
Post*fidelity ^b^	0.35	0.16–0.76	0.01
Random Effect	Variance	St. Error	
Home	0.2110	0.1269	

^a^During the study time period the MDS 3.0 was implemented. During implementation nursing homes were given leeway in documenting status. This resulted in a high number of missing observations for variables such as urinary incontinence and presence of catheter. Sensitivity analyses were performed to determine the overall effect on the models by excluding this variable; the results remained robust

^b^This interaction term reflects the difference in changes from pre to post comparing fidelity group to the non-fidelity group, so it is our main interest

## Discussion

When used, the decision-making aid significantly reduced antibiotic treatment for ASB. In the homes that implemented the decision-making aid with fidelity, the results indicated that the likelihood that an antibiotic prescribed for a suspected UTI would occur without symptoms dropped significantly in the post-intervention period.

In a number of ways, these results go beyond those of Loeb, Bentley, Bradley, and colleagues’ trial [2] aimed at reducing antibiotic treatment for suspected ASB [19]. That study found that The Loeb intervention did not significantly reduce overall antimicrobial use. It did, however, reduce the proportion of total antimicrobials prescribed for suspected urinary tract infection among implementing homes. Unlike the Loeb trial, this manuscript does not comment on whether total antibiotic use at these facilities was affected, as data on total use was not collected. As such, we can only discuss changes in antibiotic use for suspected UTIs.

The researchers selected nursing homes for inclusion in the study in a manner that created a study sample profile consistent with a “typical” sample of U.S. nursing homes, with a bed size between 80 and 120 beds, and a greater number of for-profit ownerships. Each of our three groups included homes with a variety of ownership arrangements, though the majority in each group operated as for-profit enterprises. Even with this consistency, the rate of antibiotic prescriptions for suspected urinary tract infections varied greatly between groups in the pre-implementation period, from approximately 4 to between 11 and 13 per 1,000 bed days of care, with the low intensity group having more prescriptions for suspected ASB than the high intensity group. The number of active physicians also varied considerably. Some homes had only one to three physicians responsible for residents, while others had more than a dozen. At this point, it is unclear what factor, such as variation in resident case-mix, could explain the considerable variation in antibiotic prescribing for ASB among the study nursing homes. Future studies should include information on all residents in the nursing homes, rather than only those residents listed in the infection log (see Recommendations).

Interpretation and application of the study findings should take into consideration the small study sample size (12 homes with 669 prescriptions in total), regional differences (all the homes were in Texas), and reliance on chart abstraction. The inherent nature of the study design, as a quasi-experimental nonequivalent control group design, does not control all threats to internal validity. In this era of focusing on simultaneously reducing health care costs while improving quality of care, medical chart reviews emerged as a relatively cheap (and thus common) tool for assessing quality of health care. While efficient, chart reviews are also potentially problematic. Chart reviews may be subject to bias based on characteristics associated with the recorder and the purpose of the chart. For example, a rushed scribe or variable reimbursement rates can lead to inadvertent and advertent (like up coding) errors. A study comparing chart abstraction with standardized patient reports found that chart reviews underestimated quality of care compared to the standardized patient reports for diabetes, COPD, coronary artery disease, and low back pain [20]. This underestimation was due to the standardized patients reporting better quality of care than was noted in the charts. While it is possible that prescribing physicians did not document signs and symptoms of actual UTIs (leading to an underestimation of appropriate prescriptions), we believe our chart review data were robust. Our findings are consistent with previous reports of ASB in nursing homes [15]. Furthermore, in the Luck et al. article, the majority of discrepancies between the chart reviews and the standardized patient reports were due to physicians charting actions that did not occur, rather than omitting information. Thus, if our chart review data is biased, we have reason to believe it would be biased in the direction of overestimating appropriate antibiotic use, not underestimating appropriate antibiotic use. Such an instance would work to strengthen our conclusions, not diminish.

This study also found that nursing home residents who possess the ability to communicate were more likely to receive an antibiotic prescription for ASB. This runs contrary to previous literature, in which diminished communication capacity is associated with an increased likelihood of receiving an antibiotic under a “better safe than sorry” rationale [21]. The MDS 3.0 was released during the implementation phase of our study. During that time, nurses were allowed, per CMS guidelines, a great deal of flexibility in documenting certain conditions. The flexibility gave nurses assessing residents with the MDC 3.0 the latitude to documents items related to these conditions as “not applicable”. One such condition was catheterization. As a result, our data contained an usually high number of “not applicable” responses for the question of whether or not a resident had an indwelling catheter. The high number of “not applicable” responses is potentially related to both the ability to communicate and whether or not a prescription is written for ASB, as the criteria for appropriate prescribing for residents with catheters (who are more likely to be unable to communicate) is less stringent then the criteria for residents without catheters) [22]. This may explain our results regarding communication, which diverge from our original pilot study (in which we found that cognitive impairment was associated with increased likelihood of a prescription for ASB) [15].

The decision-making aid was based on a set of best practices criteria that were formulated without a systematic evidence review. However, this form is intended for nurses to use to communicate tool with the prescribing provider, not as a diagnostic tool. Nurse communication of signs and symptoms can play a large role in provider prescribing practices [23] It is possible, however, that the decision aid affected the documentation of signs and symptoms. In this case, the decision aid led to better documentation of appropriate cases, rather than a reduction of prescriptions for inappropriate cases. While there was an overall reduction in the number of antibiotic prescriptions written overall between the pre intervention and the post intervention period (a 10 % reduction from 355 to 314), it is impossible to determine with the current data if this drop is due to the decision aid or an outside factor (such as resident flow to or from nursing homes). Likewise, it is impossible to determine the actual drop in antibiotic prescriptions for ASB without inclusion of those residents with ASB who did not receive an antibiotic prescription. This weakens our proposition that when used, the decision making aid reduced prescriptions for ASB. Future research should also include residents who did not receive a prescription to distinguish between improved documentation and improved prescribing practices [24]. In addition, because the sample is prescriptions and not residents, it does not include residents who had ASB and did not receive a prescription, nor does it include residents with the symptoms of a UTI who did not receive a prescription. As a result, we cannot determine the percentage of residents with ASB who receive an antibiotic prescription. Nonetheless, the odds ratio estimates from our multivariate analysis provided valid estimates for the odds ratios of getting a prescription for ASB comparing one level of the covariate to the baseline level.

## Conclusions

Although the decision-making aid (when used) reduced unnecessary antibiotic use during the study, in the real world of nursing home operations, it did not become embedded in the everyday operations of the study nursing homes. The four homes that implemented the decision-making aid with fidelity eventually stopped using it. The result of the facility-level analyses is yet another reminder that quality improvement interventions face significant challenges in nursing homes. Such interventions often work as promised, but they are quickly overwhelmed by competitive resource needs inherent in the day-to-day operations of nursing homes · These two findings—the success of the decision-making aid in promoting appropriate antibiotic stewardship but difficulty in adhering to the aid—suggests that future work in this area focus on methods to sustain the initial usage of the decision-making aid and its impact in an environment that does not easily adapt to new systems.

Recommendations for future studies and policies include abstracting data on all nursing home residents in study homes, not just those residents with antibiotic prescriptions or those listed in the infection logs. This will help paint a fuller picture of prescribing practices by allowing researchers to ascertain the conditions under which clinicians chose not the prescribe an antibiotic. For those interested in implementing antibiotic stewardship programs in nursing homes, we recommend dedicating resources to ensuring uptake of any program. When implemented, our decision making aid assisted in improving antibiotic stewardship in the study homes. However, homes either never fully implemented the aid or were quick to abandon the aid post implementation. Discussions with study nursing homes on this process lead to a number of recommendations for enhancing uptake and ‘stickiness’ of the aid. Those interested in implementing antibiotic stewardship programs should consider: identifying or developing an antibiotic stewardship ‘champion’ to spearhead implementation and follow up on progress; creating protocols to prevent the program from getting lost or forgotten when staff turnover; charting antibiotic use to a) show that antibiotic stewardship is needed, and b) show results of implementing an antibiotic stewardship program; and educating staff, residents, and family members on evidence-based criteria for appropriate antibiotic use.

### Availability of data and materials

The dataset supporting the conclusions of this article may be available upon request. Contact Mcmaughan@sph.tamu.edu for further information.

## Additional file

Additional file 1: (DOCX 33 kb)

## Abbreviations

ASB: asymptomatic bacteriuria
CDI: clostridium difficile infections
MDS: minimum data set
MRSA: staphylococcus aureus
SBAR: situation, background, analysis, and recommendation
UTI: urinary tract infection
