# Supplementary material for: Impact of a decision-making aid for suspected urinary tract infections on antibiotic overuse in nursing homes
Source: BMC Geriatr. 2016 Apr 15;16:81. doi: 10.1186/s12877-016-0255-9 (PMC4833907; doi:10.1186/s12877-016-0255-9)
Supplement: Additional file 1: — (DOCX 33 kb) [file 12877_2016_255_MOESM1_ESM.docx]

Appendix A.

**Suspected UTI /Fax order/Protocol**

ABC Nursing Home

123 First Street

Hello, TX 12345

Resident Name _________________________ Physician/NP/PA _________________________

Nurse ________________________________ Physician/NP/PA phone/fax _________________

Facility Phone/Fax __________________________ Date/Time ___________________________

How was information provided to clinician? : □ Phone □ Fax □ In Person □ Other __________

**S – Situation (Use this information to complete Section A&R prior to call)**

___ I am contacting you about a suspected UTI for above resident.

**Current Assessment (check all that apply):**

- Increased Urgency
- Increased frequency
- Hematuria
- Rigors (shaking, chills)
- Delirium (sudden onset of confusion, disorientation, dramatic change in mental status)

**Vital Signs:** BP _____/______ Pulse ________ Resp. rate ________Temp.________

**Resident complaints (check all that apply):**

- Dysuria (painful, burning, difficult urination)
- Suprapubic pain
- Costovertebral tenderness (flank pain/tenderness)

**Recent Urinalysis Results (Within the last 10 days) If Available:**

UA results that were obtained on ___________ (date) due to _______________________ (reason).

The results □ accompanying this communication □ are as follows: ______________________________________

**B – Background**

Indwelling catheter: □ NO □ YES

Incontinence: □ NO □ YES If yes, is this new/worsening? □ NO □ YES

Active diagnoses (especially, bladder, kidney/genitourinary conditions):

Specify:____________________________________________________________________________

Advance directives for limiting treatment (especially antibiotics): □ NO □ YES

Specify:____________________________________________________________________________

Medication Allergies: □ NO □ YES

Specify:____________________________________________________________________________

The resident is on: Warfarin (Coumadin™) □ NO □ YES

The resident is diabetic: □ NO □ YES

[Nursing Home Name] _______________________Facility FAX# __________________________

Resident Name _________________________

**A – Assessment (check boxes and determine recommendation prior to call)**

**Resident without indwelling catheter:**

□ Acute dysuria alone;

**OR**

□ Single temperature of 100◦F (38◦C), multiple at 99◦F (37◦C) or above, or 2◦F (1◦C) degrees greater than baseline AND at least one new or worsening of the following:

□ urgency □ suprapubic pain

□ frequency □ gross hematuria

□ costovertebral angle tenderness

□ new/worsening urinary incontinence

**Resident with indwelling catheter:**

□ fever of 100◦F (38◦C)or 2◦F (1◦C) greater than baseline

□ new costovertebral tenderness

□ rigors

□ new delirium

□ hypotension

**Any one of the above present**

□ **Protocol criteria ARE met.**

According to our understanding of best practices and our facility protocols the resident may have a urinary tract infection and need a prescription for an antibiotic agent.

□ **Protocol criteria are NOT met.**

According to our understanding of best practices and our facility protocols, the information is insufficient to indicate an active urinary tract infection. The resident does NOT need an immediate prescription for an antibiotic, but may need additional observation.

Yes

Yes

No

**R - Recommendation**

No

**Staff:**

**Please Check Box for Course of Action Recommended**

**Physician/NP/PA Orders:________________________________________________**

How were orders provided by clinician? : □ Phone □ Fax □ In Person □ Other

□ Ordered U/A (with C&S if indicated)

*Would you like to initiate any of the following?*

□ Encourage 4 ounces of cranberry juice TID.

□ Record fluid intake

□ Assess vital signs, including temp; every ___ hours for ____ hours

□ Notify Physician/NP/PA if symptoms worsen or if unresolved in ____ hours

□ Other: ___________________________________________________________

□ Initiate the following antibiotics

Specify: _________________________________________________________________________

□ Other ________________________________________________________________________

**Physician/NP/PA signature** ____________________________date/time_____________________

Telephone order received by ____________________________date/time_____________________

Family/POA notified (name) ____________________________date/time: ____________________
